# A Treatise on Poisons

**Published:** 1845-04

**Authors:** 


A Treatise on Poisons. By *Robert Christison*, M.D. F.R.S.E. Fourth Edition. Black, Edinburgh. 1845. Octavo, pp. 986.

Traite de Toxicologie. Par *M. Orfila*. Fourth Edition. Paris, 1843. 2 Tom. 8vo, pp. 1465.

Principles of Forensic Medicine. Part III. Toxicology. By *W. A. Guy*, M.B. Renshaw, 1844. 8vo, pp. 170.

A Toxicological Chart. By *W. Stowe*, M.R.C.S. Tenth Edition. Highley, 1845.

It says much for the progress and stability of Toxicological Science, that while the fourth editions of the two first-named celebrated works contain a large addition of new facts, and many improvements upon former modes of investigation, their authors find it necessary to abandon no important principles heretofore laid down. Readers will here find a record of every, even the most recent, improvement, whether as regards the detection of the poison or the administration of its antidote. But, although the attentive perusal and frequent consultation of M. Orfila's Treatise, rich as it is in its original observations, ingenious processes, and collection of innumerable experiments, is essential to all engaged in the study of this branch of medical science, yet for the practical man we consider Dr. Christison's book a very superior guide, by reason of its greater perspicuity, the more complete view of the entire subject which it embraces, and the constant references to useful cases, sources of farther information, and important trials with which it teems.

In the present article we intend to notice the subject of General Poisoning at some length, availing ourselves especially of Dr. Christison's observations, which are far more complete upon this subject than those of Orfila; and afterwards to allude to some of the more important particular poisons.

### Physiological Action of Poisons.

1. *The Mode of Action.*—The belief that poisons produce their effects through the medium of the nervous system, *i. e.* by *sympathy*, was at one time generally entertained. The discoveries of Magendie upon venous absorption, and the experiments of later enquirers, have demonstrated that, in the great majority of instances, *absorption* becomes the active agent in influencing the remote organs. Indeed Mr. Blake endeavours, in his papers in recent volumes of the Edinburgh Journal, to prove this to be their exclusive mode of action, even in cases in which death seems to occur almost instantaneously. Dr. Christison, while admitting that the explanation of the *modus operandi* of certain of the poisons upon the principle of sympathy is somewhat shaken, is unable, in the present state of the inquiry, to concur with Mr. Blake in his opinion that it is never admissible.

"Mr. Blake, who is altogether opposed to the occurrence of nervous transmission in the instance of any poison, has found that ammonia, injected into the jugular vein of a dog, was indicated in its breath in four seconds; and that chloride of barium or nitrate of baryta, introduced into the same vessel, could be detected in the blood of the carotid artery in about sixteen seconds in the horse, in less than seven seconds in the dog, in six seconds in the fowl, and in four seconds in the rabbit. These interesting discoveries will not however absolutely destroy the conclusiveness of all the facts quoted above in support of the existence of a sympathetic action. For example, they do not shake the validity of those observations, in which it appeared that an interval inappreciable, or barely appreciable, elapsed between the application and action of hydrocyanic acid and of conia. Mr. Blake indeed denies the accuracy of these observations, insisting that, in those he made himself with the most potent poisons, he never failed to witness, before the poison began to act, an interval considerably longer than what had been observed by others, and longer also than what he had found sufficient for the blood to complete the round of the circulation; that, for example, the *wourali* poison, injected into the femoral or jugular vein, did not begin to act for twenty seconds, conia and tobacco for fifteen seconds, extract of nux vomica for 12 seconds; and that hydrocyanic acid dropped on the tongue did not act for eleven seconds if the animal was allowed to inhale its vapour, and not for sixteen seconds if direct access to the lungs was prevented by making the animal breathe through a tube in the windpipe. But Mr. Blake cannot thus summarily rid himself of the positive facts which stand in his way. Duly weighed, the balance of testimony is in favour of those whose accuracy he impugns. For, in the first place, they had not, like him, a theory to build up with their results, but were observing, most of them at least, the simple fact of the celerity of action. Then, their result is an affirmation or positive statement, and his merely a negative one. They may perfectly well have observed what he was not so fortunate as to witness. And lastly, it is not unreasonable to claim for Sir B. Brodie, Dr. Freer, Mr. Macaulay, and Mr. Taylor, all of them practitioners of experience, the faculty of noting time as accurately as Mr. B. himself. As for my own observations, I feel confident they could not have been made more carefully, and that I had at the moment no preconceived views which the results upheld, but, if any thing, rather the reverse. It is impossible therefore to concede, that Mr. Blake's inquiries, merely because they are at variance with prior results, apparently not less precise and exact than his own, put an end to the argument which has been drawn, in favour of the existence of a sympathetic action, from the extreme swiftness of the operation of some poisons. At the same time, on a dispassionate view of the whole investigation, it must be granted to be doubtful, whether this argument can be now appealed to in its present shape with the confidence which is desirable. And on the whole, the velocity of the circulation on the one hand, and the celerity of the action of certain poisons on the other, are both of them so very great, and the comparative observation of the time occupied by the two phenomena respectively becomes in consequence so difficult and precarious, that it seems unsafe to found upon such an inquiry a confident deduction on either side of so important a physiological question as the existence or non-existence of an action of poisons by sympathy."

2. *The Action of Poisons through Absorption.*—However the action of a few poisons through the medium of the nervous system may be disputed, there can be no doubt the majority act by being absorbed into the blood. Various familiarly-known experiments amply prove this. Addison and Morgan have endeavoured to show that the poisons so admitted into the blood do not act by being transmitted with that fluid to the organs affected by them, but by the influence they exert upon the nerves of the bloodvessels. This opinion has been much opposed, and may now be considered as disproved, the experiments adduced in support of it being either erroneous or fallacious. Thus, numerous observers have proved that poisonous substances absorbed by the veins of the part to which they are applied may be detected in the tissues of distant organs. Mr. Blake has shown that poisons produce their effects more quickly when injected into the aorta than into the venous system; and that their effects are rapid in proportion to the velocity of the circulation of the animal employed for the experiment.

"On the whole, then, it may be considered as well established, that probably all, but certainly some, poisons,—of the kind whose topical action does not consist in causing destruction or inflammation of the textures to which they are applied,—produce their remote effects solely by entering the blood, and through its means impregnating the organs which are acted on at a distance. And farther, if this doctrine be admitted as established, it may also be allowed, that many poisons which do cause topically destruction or inflammation, and remotely the usual sympathetic effects of these changes of structure, also possess the power of affecting distant organs through the medium of the blood."

M. Orfila quotes the conclusions of Blake, with great approbation, and enumerates the following circumstances as exerting an influence upon the absorption of poisons.

"1. Loss of blood favours their absorption. 2. It may be stated as a general rule that the absoption of any substance soluble in a fluid, is much more rapid so dissolved than when applied in the solid state. Thus, a solution of the aqueous extract of opium will produce fatal effects a few minutes after its application to the cellular tissue of the thigh, while the same extract applied in its solid state acts much more slowly. 3. It would be a mistake, however, to deny the absorption of some poisons possessed of little solubility. Thus arsenic, which is so slightly soluble in water, is absorbed with rapidity. 4. Poisons, externally applied, are usually most rapidly absorbed in proportion as the part is abundantly supplied with lymphatic and venous absorbents. Nevertheless, the locality in some cases does not seem to exert any effect; thus, whether arsenic be applied to the cellular tissue of the back or of the inner side of the thigh of a dog, death occurs in both cases at the end of from three to six hours. It may even happen that the dog to whose back it has been applied shall die first. The same quantity of corrosive sublimate, on the other hand, which, applied to the thigh of a dog, will kill it in from fifteen to twenty-four hours, will not cause its death until six or seven days, if applied to its back. 5. The absorption of certain poisons may take place, although they are not brought immediately in contact with the animal tissues. Sal ammoniac or arsenic is absorbed when applied to the cellular tissue in linen bags. 6. There are some poisons which are entirely absorbed, and of which no trace can be found upon examining the part to which they have been applied. On the other hand, a great number are only partially absorbed, and large proportions are found upon the part where they have been placed. Thus, if we apply to the cellular tissue 8 parts of the powder of a vegetable poison, after the death of the animal, 5, 6, or 7 of these may remain—the active portion seeming alone to have been absorbed. But, when the essentially poisonous portion of a vegetable powder is applied, the whole is yet not absorbed, because life is promptly destroyed, and absorption ceases with it."

3. *The Discovery of Poisons in the Blood and Tissues* has only been accomplished of late years. Many circumstances prohibited it. Thus, in the case of very active poisons, the small quantity employed defied detection by the ordinary means of analysis. Various poisons are removed before death by passing off with the urine, &c. Several of the poisons do not seem to remain in the blood, but become deposited in particular organs, as the liver, which have only of late been submitted to examination. Some poisons, again, are rapidly decomposed on entering the blood. Thus, Dr. Christison was unable to discover oxalic acid in the blood of the *vena cava* of a dog killed in 30 seconds by the injection of $8\frac{1}{2}$ grains into the femoral vein.

"The improvements that have been lately made in the methods of analysis for the detection of poisons in a state of complex mixture with organic substances have done away with a great part of the obstacles which prevented a thorough inquiry as to the existence of poisons in the blood and textures of the body. Some important researches of this kind were referred to in the last edition of the present work; and since then many additional facts of equal variety and precision, have been communicated by different observers, but especially by Professor Orfila. Under the head of each poison, an account will be given hereafter of the evidence in support of the discovery of it by chemical analysis in the blood, textures, and excretions. In the present place it is sufficient to state in general terms that the evidence is quite satisfactory in the instances of iodine, sal-ammoniac, oxalic acid, nitre, sulphuret of potassium, arsenic, mercury, copper, antimony, tin, silver, zinc, bismuth, lead, hydrocyanic acid, cyanide of potassium, carbazotic acid, sulphuretted hydrogen, camphor, and alcohol."

4. *The Organs affected by the remote Action of Poisons.*—Poisons do not, as sometimes stated, affect the general system; and although a few, as oxalic acid, arsenic, and mercury, affect a great number of organs of the body at once, others affect some one important organ in particular. The researches of Mr. Blake have thrown new light upon this as well as upon so many other points relating to the action of poisons. Some affect solely or chiefly the *Heart*. Brodie found that the injection of *tobacco* or *upas antiar* into any part of the body totally paralysed the action of this organ, so that galvanism produced no excitement. Campbell observed the same as regards *arsenic*, and Christison as regards *oxalic acid*—although the other voluntary and involuntary muscles still retained their irritability. Mr. Blake has shown the similar action of various other substances when injected into the jugular vein, as salts of magnesia, zinc, copper, lime, strontia, baryta, lead, silver, ammonia and potash, oxalic acid and digitalis. Other poisons affect the *Lungs*, though probably none do so alone. Thus, tartar emetic and corrosive sublimate induce appearances of inflammation. Mr. Blake has shown that several poisonous substances, which when injected into the veins affect the heart, do not produce any effect upon the lungs, such as oxalic acid, and the salts of magnesia, lime, zinc, copper, ammonia, potash, and strychnia. "Others, however, as the salts of strontia, baryta, lead, and silver, as well as digitalis, all of which powerfully affect the heart, and, in addition to these, the salts of soda, which have no action at all upon the heart, and hydrocyanic acid, tobacco (?), and euphorbium, which influence it feebly, and even dubiously,—produce, when injected into the jugular, obstruction of the capillaries of the pulmonary circulation, and consequently asphyxia." Many poisons operate upon the *Brain*, as the narcotics and most narcotico-acrids, frequently, however, affecting other organs, as the spine and heart, at the same time.

Mr. Blake has shown that the influence exerted upon the brain by the injection of certain substances into the veins, as the salts of soda, are secondary, and dependent upon the obstructed condition of the pulmonic circulation ; but, usually, the effect produced upon this organ, as by opium, is direct—the circulation and respiration becoming only secondarily affected by reason of the annihilation of the functions of the nervous system. Some poisons act specifically upon the *Spinal Cord,* as strychnia, conia, and the wourali.

"Conia, the active principle of hemlock, according to my own researches, produces in the lower animals, however introduced, gradually increasing paralysis, without insensibility or delirium, and without the circulation or respiration being for some time affected, till at length death takes place from stoppage of the breathing by palsy of the respiratory muscles ; and, after death, the heart continues beating vigorously, the muscles contract when irritated, and arterialization of the blood in the lungs may be kept up long by maintaining artificial respiration. In this instance it would appear, that the first effect is arrestment of the function of the spinal cord ; that the paralysis does not depend upon a direct action on the muscles ; and that neither the brain, heart, or lungs can be influenced, except secondarily, through the consequences of general muscular paralysis. Many poisons which act on the brain also act on the spinal cord."

Mr. Blake's experiments seem to show that certain poisons possess the power of impeding or arresting the *general capillary circulation,* acting on this in common with the pulmonic capillaries, or in a few instances affecting it independently. The various organs not essential to life may be acted upon by poisons, and perhaps the spleen and pancreas are the only considerable organs which are not affected by some poison or other.

5. *Causes modifying the Action of Poisons.*—The action of poisons differs according to the operation of several circumstances. Of these the principal are—(1) *Quantity.* For example, oxalic acid, according to its dose, may produce its local effect upon the stomach, or affect the heart, the spine, or the brain. So a small dose of arsenic may give rise to gastritis, a large one may produce speedy death by its action upon the heart. (2) *Aggregation.* The more minutely a poison is divided the more rapidly and certainly it acts. Thus several, which in their solid state produce little effect, produce speedy death given in solution. (3) *Chemical Combination.* This is sometimes only influential by augmenting solubility. Morphia almost inert, because insoluble, becomes active by the acquisition of solubility upon its junction with acids. Two general laws may be stated ; one, that *poisons which only act locally, have their action much impaired or even neutralized, in their chemical combinations :* the other, *that the action of poisons which operate by entering the blood, although it may be somewhat lessened, cannot be destroyed or altered in their chemical combinations.* Sulphuric acid and the fixed alkalis are examples of the first of these, and morphia of the second. Mr. Blake has endeavoured to prove that *salts of the same base produce the same actions,* independently of the acids with which they are combined ; and he considers it probable that those which *are isomorphous, or possess the same crystalline forms, are closely allied in action.* (4) *Mixture.* This may influence by dilution which generally, although not with a powerful irritant, lessens the action ; or by offering a mechanical impediment to the action of poison on the tissues, as when the substance mixed with it is in fine powder. (5) *Difference of Tissue.* Poisons, with the exception of the corrosive and irritant, manifest a difference in their action according to the tissue they are applied to, acting " least energetically on the skin, more actively on the alimentary mucous membrane, still more so on the serous membranes, and most powerfully of all when introduced directly into a vessel." Their action upon the pulmonary mucous membrane takes place almost as rapidly as when introduced into a vein. Poisons which act upon the sentient extremities of nerves do not act at all upon the cut surface of the brain or nerves, or upon any part of the course of the latter. It is not only as regards the rapidity of action that different tissues influence the effects of poisons. Thus a poison, as of a viper, which injected into a vein proves rapidly fatal, if swallowed in large quantity is harmless.

" Differences in the absorbing power of the tissues cannot explain these facts. The only rational way of accounting for them is by supposing that a part of the poison is decomposed,—the change being greatest where the absorption is slowest, and the power of assimilation strongest, namely in the stomach,—and least where absorption is quickest and assimilation almost wanting, namely in a wound. This explanation derives support from the different effects of change of tissue on poisons of the different kingdoms. Mineral poisons are least, and animal poisons are most, affected in their action by differences of tissue, while vegetable poisons hold the middle place; an arrangement which coincides with the respective difficulty of decomposition among mineral, vegetable, and animal substances generally, whether under physical or under vital processes. These views regarding the decomposition of poisons, were suggested to me in 1823, by my friend Dr. Coindet, Jun. of Geneva."

(6) *Differences of Organ* are not entirely referable to differences of tissue: but depend much upon the importance of the organ in the general economy. (7) *Habit* and *Idiosyncracy.* The effect of *habit* in lessening the energy of the action of some poisons is familiarly known. The poisons which act upon the brain and nervous system, and induce narcotism, as opium and alcohol, are those chiefly observed to be so influenced. Even with regard to these, habit usually only diminishes their immediate effects, disease or a predisposition to it, being engendered, except seemingly in the case of tobacco, by their long-continued use. Of another class of poisons Dr. Christison observes—

" But as to *inorganic poisons* that enter the blood, habit certainly does not diminish, probably rather increases their power. There is no satisfactory evidence, that a person by taking gradually-increasing doses of arsenic may acquire the power of enduring a considerably larger dose than when he began. On the contrary, the stomach rather becomes more tender to the subsequent dose by each repetition. I have little hesitation in avowing my disbelief of the alleged cases of arsenic eaters and corrosive sublimate eaters, who could swallow whole drams at once with impunity. Some have expressed surprise at this statement having been made in former editions of this work, when there is such authority as Byron, Pouqueville, &c. for the hacknied story of Solyman, the sublimate eater of Constantinople, who lived to the age of 100, eating a drachm of the sublimate daily. I must avow, however, that such reporters of a feat so very extraordinary, and where deception was so highly probable, are to me no authority at all.

"In the relative influence of habit on poisons of the three kingdoms of Nature, a new argument will be discovered for the opinion given above, respecting the partial decomposition of organic poisons in some of the tissues. In fact, this partial decomposition accounts very well for the effect of habit. The effect of habit is probably nothing more than an increased power acquired by the stomach of decomposing the poison,—just as it gradually acquires an increased facility in digesting some alimentary substances which are at first very indigestible."

*Idiosyncracy* generally increases the activity of poisons, and renders numerous articles of diet, &c. poisonous to certain individuals which are usually harmless. The examples of this, as regards opium, calomel, shell-fish, mushrooms, &c. &c. are familiar to all. The only instances in which idiosyncracy would seem to decrease the influence of poisonous substances are mercury and alcohol, and, in some very rare cases, opium.

(8.) *Diseased States of the Body*, like the effect of habit, often impair the activity of poisons. In various forms of fever, there is a diminished susceptibility to the action of mercury, and in others to the action of alcohol. In severe dysentery or epidemic cholera, hæmorrhage, &c. a person may take opium to an extent that would prove fatal in health. Tetanus is remarkable for the insusceptibility to the action of the most potent drugs which it engenders. In mania and delirium tremens immense doses of narcotics may be endured. Hydrophobia always, and hysteria sometimes, impairs the activity of poisons.

"In the operation of this class of modifying agents it is a general law, to which there are probably few exceptions, that they chiefly affect poisons of the organic kingdoms, and the narcotics above all. At least in the instance of most mineral poisons their influence is very inferior. Their operation may be accounted for in various ways. Sometimes, as in dysentery and cholera, the poison is carried with unusual rapidity through the alimentary canal. Sometimes, again, it remains comparatively inert, because, on account of the impaired activity of absorption, it is not taken up with the usual quickness by the absorbent vessels. And sometimes, as in the instance of tetanus, mania, and rabies, the nervous system is in a state of peculiar excitement, by which the customary action of the poison is in a great measure, if not entirely, counteracted."

### General Observations upon the Treatment of Poisoning.

Dr. Christison observes that *antidotes* are of two kinds, the one altering the chemical nature of the poison before it has had time to produce its effects, the other setting up in the system a contrary action to that induced by the poison after its operation has commenced. Antidotes of the latter description, formerly believed to be common, are in fact rare in the extreme; the correction of the remote effects of lead by salivation with mercury, and those of prussic acid by ammonia, being the two chief that can be mentioned. The chemical antidotes may simply operate by neutralizing, and thus destroying, the corrosive effects of a poison, or by destroying the solubility to such an extent as to render its absorption as slight as possible. Many formerly reputed antidotes have been found by modern research not to be possessed of these necessary qualities, both of which, if possible, should exist in the substances employed.

"In recent times a new object in the treatment of poisoning has been pointed out by the discoveries made in its physiology. As it has been proved that many of the most deadly poisons enter the blood, and in all probability act by circulating with that fluid, so it has been inferred that an important object in the treatment is to promote their discharge by the natural secretions. In support of this reasonable inference, it has been lately rendered probable by Orfila, as will be seen under the head of the treatment of the effects of arsenic, that it is of great advantage in some forms of poisoning to increase the discharge of urine."

M. Orfila, speaking of the general treatment of poisoning, says:

"As I observed in my first edition, we must distinguish *two epochs* in the treatment of poisoning. 1. The poison has not been long swallowed, and it yet remains in the digestive canal. We must endeavour to prevent its action by expelling it upwards or downwards, or by combining it with a substance capable of neutralizing its poisonous properties. This object fulfilled, we meet the symptoms which have been produced by the poison by means appropriate to the particular case. 2. The poison has been long swallowed, and vomiting or purging have already occurred, so that the portion of the poison which has not acted has been expelled. It would compromise the safety of the patient if in this case we endeavoured to decompose the poison, and we must simply oppose the progress of the malady by appropriate general treatment."

Of *counterpoisons or antidotes,* which are to be employed during the *first epoch,* in conjunction with the evacuation of the stomach by emetics or the stomach-pump, M. Orfila observes:

"A substance is designated an antidote or counter-poison when possessed of the following properties. 1. It may be taken in large quantities without any danger. 2. It must be able to act upon the poison at a temperature equal to, or lower than, that of the human body. 3. Its action should be speedy. 4. It must possess the power of decomposing the poison amid the gastric and other secretions the stomach may contain. 5. In acting upon the poison, it should deprive it of all its deleterious properties. There are medicinal substances which form with poisons compounds much less poisonous than these poisons, but which are yet deleterious. These cannot be considered as *perfect antidotes,* but as medicaments which we must employ until others, capable of depriving the poison of all deleterious properties, can be discovered. We may then divide antidotes into two sections:—(1) such as *destroy completely* all deleterious qualities, as the soluble sulphates for the salts of barium and of lead, the soluble chlorides for the salts of silver, &c.: and (2) those which *notably diminish* the fatal effects of poisons, such as albumen for the salts of mercury and copper, gall-nuts for opium, &c."

### EVIDENCE OF POISONING.

This, the medico-legal portion of the subject, is treated at some length by both the authors whose works we have so often quoted, and Dr. Guy presents, in his "Principles of Forensic Medicine," a fair summary of Dr. Christison's views. Dr. Guy has been charged with having, in the composition of this concluding part of his work, made an undue use of Mr. Taylor's Manual of Medical Jurisprudence. A charge of plagiarism is a grave affair, and one not very easy to substantiate in a branch of medical science like Toxicology, where original facts added by each successive writer cannot be very numerous ; and in the present case it may have been somewhat exaggerated, and some of the proofs adduced in support of it are perhaps far-fetched. Still, we believe that no one can compare the one book with the other without arriving at the conclusion that Dr. Guy has availed himself of Mr. Taylor's labours to an extent that cannot be justified. The recent publication of the Manual, and a somewhat similar error formerly committed with respect to another work of the same author, ought to have impressed a caution which does not seem to have been observed, or have produced the imperfect reparation of a special acknowledgment in the preface to the book. If, almost immediately after the publication of a work, it is allowable to seize hold of it, and submit it to a condensing process, merely because it is the last which has appeared upon the subject, the craft of authorship, or rather of book-making, becomes an easy one indeed. The former parts of Dr. Guy's work contained much useful matter, (though not unvitiated by the same defect,) and some original observations; and it would have detracted nothing from his reputation had he candidly avowed his obligations to so skilful a toxicologist as Mr. Taylor, although the better course would certainly have been not to have incurred them to this extent.

We must however quit this unpleasing theme: and proceed to enumerate the various sources of Evidence of Poisoning.

1. *Evidence from Symptoms.*—M. Orfila offers several good general observations upon the value which should be attached to symptoms observed during life, and lesions discovered after death; some of which we extract.

"I have often in the course of this work opposed the opinion of those practitioners who believe they can, by observation of the symptoms and lesions of tissue, not only determine that poisoning has taken place, but the description of poison employed. Nevertheless I am far from asserting that it is useless to make an attentive examination of these symptoms and lesions. On the contrary, I am perfectly convinced that, in most cases, it is indispensable to take them into account, to be enabled to affirm that there has been poisoning, and that, under some circumstances, they may assist us in determining to what class the poison whose nature we may be investigating belongs. *In no case does the existence of a poison in a suspected substance alone suffice for leading to the conclusion that poisoning has occurred;* and we must necessarily join to this important element of medico-legal research the proofs derived from the symptoms, and frequently from the changes after death. It would be a grave error to suppose that it sufficed to procure a certain quantity of a poisonous substance from matters vomited or passed by stool, or even from the corpse, to be enabled to affirm that poisoning has taken place. The practical chemist, ordinarily charged with this description of analysis, should confine himself to stating that, by this or that process, he has been enabled to procure arsenic, copper, &c. The element in the inquiry which he furnishes is an important one, but it is insufficient; for, in fact, malevolence may have introduced the poison into the substances examined after death. Again, the patient may have employed shortly before death medicinal substances containing arsenic or copper, &c. in large or small doses, and a portion of these may be found either in the digestive canal or the viscera. The poison, too, may exist *naturally* in the body in small proportions, and, if care be not taken to distinguish whether that detected may have existed normally, or have been swallowed, serious error may arise. In fact, nothing is more easy than determining whether the *lead* or *copper*, the *only dangerous metals whose existence in the human body is indubitable*, which is detected, has been swallowed, or is only such as exists normally in the system."

After alluding to the diseases, such as gastritis, cholera, &c. whose symptoms resemble those occasioned by poisoining, he protests against the unfair use which is often made of the admission of such resemblance.

" It is very wrong for imprudent advocates, whether physicians or not, to seize indiscriminately every occasion which is offered them, for affording support to an accused person by affirming that any evidence drawn from the symptoms manifested by the victims of poisoning, is of no real value. Those who have had the opportunity of observing and examining patients at different epochs after poisoning, hold a different opinion, and do not allow themselves to be led away by loose generalities. * * * * * * They know that the *ensemble* of the symptoms scarcely ever, not to say never, manifest themselves in any other case than poisoning; and that consequently it is completely false to declare that they are *commonly* observed in many spontaneous diseases, as many men unacquainted with our profession, especially M. Raspail, would have us believe of late. We think it requisite to call the attention of practitioners particularly to this point, and to engage them, whenever they are witnesses of these facts, to take great care in preserving the matters vomited, the stools, and the urine of the patient. It is a fact that negligence in this particular is extreme. How frequently have we had to deplore the omission of these precautions; for is it not undeniable that the vomits, stools, and urine, would have furnished unequivocal proof of poisoning, in cases where it has been impossible to prove it by an analysis of the remaining contents of the digestive canal and other organs? It is also very important, in order to direct a suitable treatment, to be informed promptly whether poison, and what poison, has given rise to the symptoms. I am aware that there have been some very rare cases, in which persons poisoned, and even by irritant poisons, have manifested no symptoms whatever, and these too have been brought prominently forward by superficial minds, in order to diminish the value which should be attached to the symptoms which ordinarily appear. This is an error, for these cases are truly exceptional: and we could scarcely cite four or five well-attested ones in opposition to the innumerable examples of those of a contrary description.

" I cannot terminate this subject without strongly blaming the conduct of those who, summoned before the tribunals for the purpose of appreciating the degree of value to be attached to the symptoms, base their denial of the act of poisoning upon the fact, that the patients *have not manifested all the symptoms* described by authors as characterizing the description of poisoning in question. Will it be believed that, in a case of this kind, in which one of the accused avowed the crime, that one of our *confreres* maintained against me that poisoning by arsenic had not taken place, because the patient had not manifested *some* of the symptoms mentioned in my works. An objection like this possesses no validity, and should not find any favour in the eyes of the Court. Authors who describe *all the symptoms in a general manner*, or which have been observed up to the present time, in those poisoned by the same substance, do not pretend to say that the *ensemble* of these is *necessarily* to be found in every case. In giving a *resumé* of their observations they desire to enumerate all the circumstances which have been already observed, but certainly do not mean to state that all such should be found indiscriminately in every individual case. It may be believed, on the contrary, that in this respect there must be infinite varieties, according to the dose of the poison, the age, constitution, and state of health of the person poisoned, the duration of the effects, and the means employed to combat them, &c."

Dr. Christison, in reference to the doctrine now maintained by all writers on medical jurisprudence, that symptoms, however well-developed, can never justify an opinion beyond high probability, although formerly they were considered as all-sufficient, observes,—

"In laying down this doctrine medical jurists appear to me to have injudiciously confounded together actual symptoms with their general characteristics. If the doctrine is to be held as applying to the evidence from symptoms, only so far as they are viewed in questions of general poisoning—that is, as applying to the general characters merely of the symptoms,—it is deduced from accurate principles. But if it is likewise to be applied, as recent authors have done, to the actual symptoms produced by particular poisons, and in all cases whatever of their action, then it is clearly a rule liable to many important exceptions. These exceptions will be noticed under the heads of mineral acids, oxalic acid, arsenic, corrosive sublimate, nux vomica, &c. At present it is only the general characters of the symptoms, and the points in which they differ from the general characters of the symptoms of natural disease, that I propose to consider."

Of these, the following are enumerated—1. The symptoms are sudden in their appearance and rapid in their progress. 2. Regular in their increase. 3. Uniform in their nature throughout their progress. 4. They commence soon after food, drink, or medicine has been taken. 5. The symptoms appear during a state of perfect health.

These characteristics by no means apply to all poisons or to the same under all circumstances, nor do they always present points of distinction sufficiently marked to render the diagnosis between the symptoms of poisoning and those occasioned by various diseases, certain. Nevertheless, they are often of great corroborative utility, and their consideration can never be neglected by the medical jurist.

"For, in the first place, they are of great value as generally giving him the first hints of the cause of mischief, and so leading him to search in time for better evidence. Next, they will often enable him to say that poisoning was possible, probable, or highly probable; which, when the moral evidence is very strong, may be quite enough to decide the case. Thirdly, although they can never entitle him to say that poisoning was certain, they will sometimes enable him to say, on the contrary, that it was impossible. And to conclude, when the chemical or moral evidence proves that poison was given, the characters of the symptoms may be necessary to determine whether it was the cause of death.

"As the last statement is one of consequence, and yet has been overlooked by some authors on medical jurisprudence in this country, it may be illustrated by one or two comments. It does not follow, because a poison has been given, that it is the cause of death; and therefore, in every medico-legal inquiry, the cause of the first symptoms and the cause of death should be made two distinct questions. The question, whether a poison, proved to have been administered, was the cause of death, is to be answered by attending to the second and third characteristics mentioned above, and considering whether the symptoms went on progressively increasing, or altered their nature during the patient's illness, and whether the alteration, if any, was such as may occur in the case of poisoning generally, or of the special poison given."

Dr. Christison illustrates this point by referring to a case in which a girl poisoned by arsenic recovered from its effects, and then died with symptoms of obscure general fever.

2. *Evidence from Morbid Appearances.*—Some of these, to which considerable weight was formerly attached, as lividity of the skin, or early putrefaction of the body, are now not deemed of any importance. Even others, such as an inflamed condition of the alimentary canal, or congestion of the brain, are not invariably present, and are not distinguishable from similar appearances caused by disease. They may afford important corroborative evidence however, although taken alone they are of no avail. A few poisons, as the mineral acids, produce some characteristic appearances; and, where there is no doubt of poison having been given, the appearances may be of avail in determining whether death has been caused by it. Dr. Christison draws attention to the fact, that the presence of certain appearances of natural disease must not prevent our investigating whether other causes, such as poison, may not have proved the actual cause of death; and mentions instances of extensive disease of a latent character having been found in the bodies of persons who had yet died poisoned.

"The conclusions to be drawn from these facts are that, at all events, the medical inspector, in a question of poisoning, must take care not to be hurried away by the first striking appearances of natural disease which he may observe, and so be induced to conduct the rest of the examination superficially; and likewise, that he should not so frame his opinion on the case, as to exclude the possibility of a different cause from the apparent one, unless the appearances are such as must necessarily have been the cause of death. * * * * * * At present no more need be said, than that the inspector should be particularly on his guard in those cases, in which the appearances, though belonging to the effects of a deadly disease, are trifling; and still more in those in which the appearances, though great, belong to the effects of a disease, whose whole course may be latent. And I may add, that, from what I have observed of medico-legal opinions, the caution now given is strongly called for."

M. Orfila thus sums up the results of his observations upon this branch of the subject:—

"(1) Irritant poisons and a portion of the narcotico-acrids, almost always produce inflammation in one or other portion of the alimentary canal, when taken in a sufficient dose; but this is not the case with the narcotics and the remainder of the narcotico-acrids. (2) It has, however, been completely proved, that, under certain circumstances, some of the irritant poisons have caused death without leaving the slightest trace of alteration in the digestive canal. (3) In a case where, after the early death of an individual suddenly attacked with grave symptoms, the alimentary canal is found inflamed, corroded, ulcerated, &c., we must strongly suspect poisoning, because this *ensemble* of symptoms and lesions is seldom observed in spontaneous disease. (4) Generally speaking, lesions of the lungs, brain, heart, and other organs, are capable of being produced by a too great number of causes, to allow of their being adduced as proofs of poisoning. (5) The physician cannot affirm that poisoning has occurred until he has indubitably proved the existence of the poisonous substance, either by chemical analysis, or by its physical properties. (6) Where this substance cannot be discovered, and the symptoms and lesions of tissue are analogous to those caused by poisoning, he can only state that the *probabilities* are in favour of this having been the cause of death."

3. *Evidence from Chemical Analysis.*—This is justly deemed the most satisfactory of all the proofs that can be offered, and the detection of the poison in the stomach or intestines, blood, vomits, or stools, &c. usually supersedes the necessity of any additional assurance. Corroboration, however, by an accurate relation of the symptoms and morbid appearances, is always desirable. It may even be essential in cases in which the poison is supposed to have been introduced into the parts examined subsequently to death, and for the decision of the question whether the poison, proved to have been administered, has been the cause of death or not. However desirable the proof from chemical evidence may be, it is not always to be attained : for several causes may have removed the poison beyond the reach of investigation, death having nevertheless been caused by its instrumentality. Thus, (1) it *may have been discharged by vomiting and purging*. In the body of a man who died eight hours after taking an ounce of arsenic, none could be detected chemically, and in another case, where death resulted in five hours from a solution of arsenic, careful analysis could detect none of the poison. Such cases are the more interesting because it is remarkable for how long a period arsenic will frequently resist the efforts of the stomach to dislodge it entirely, it having been found in that organ after two days of incessant vomiting. Modern improvements in analysis prove that poisons are often retained in the stomach a much longer period than was heretofore suspected. (2) The *poison may have been all absorbed*. This is generally, but not always, the case with laudanum or opium. (3) The *excess of the poison may have become decomposed*. Opium has been in this way digested, and some mineral poisons decomposed in the stomach, still, however, in the last case, remaining amenable to analysis. The changes attendant upon the putrefaction of the body may also give rise to decomposition of the poison. Thus many poisons existing in the stomach at the time of death cannot be detected some time after interment. This is not so much the case with mineral poisons, which have been detected months and years after burial. The researches of Orfila have proved that the prevention of the detection of poisons by their decomposition during the decay of the animal matters around them is much less common than might be expected. (4) Poisons which have been absorbed may be *removed by excretion*.

In reference to this part of our subject M. Orfila presents some interesting remarks upon the following question. "*Is it necessary, in order to establish the fact of poisoning, to recover a determinate quantity of the poison employed, or is it sufficient to prove that this substance exists in any proportion whatever?* He observes that of late, since his improved procedures have so frequently revealed very small quantities of arsenic, antimony, copper, &c. many practitioners, little accustomed to toxicological research, have accused him of temerity in drawing conclusions from quantities so insignificant. In answer to this opinion, which if admitted, would seriously compromise the interests of society, he states, 1st. *In certain cases of poisoning by mineral substances, susceptible of detection by re-agents, the experimenter is unable to detect the smallest atom of the poison.* Suppose, for example, that the stomach has been completely cleared out by vomiting, and the matters vomited have not been preserved. No poison will be detected although poisoning yet has taken place. So, if the research is pursued for the portion that has been absorbed, the success which will attend it depends upon when it is commenced.

"Let several dogs be poisoned by the application of arsenic or tartar-emetic to the cellular tissue of the inner portion of the thigh, and leave some of these animals to their fate. After their death, which will occur in 30 or 40 hours, there will be no difficulty in obtaining notable quantities of arsenic from their viscera. Let others of the poisoned dogs, on the other hand, be submitted to an abundant *diuretic medication,* and if we can cause them to urine abundantly for three or four days they will not die. If we kill them about the ninth or tenth day, not the slightest trace of the poison will be discoverable in their viscera, while their urine during the whole time it has flowed since they were poisoned has afforded appreciable quantities of it. It may then happen that an individual has taken a certain quantity of poison, insufficient to kill him in a few hours, that he manifests symptoms of poisoning during 8, 10, 12, or 15 days, that during this time the poison becomes entirely expelled by vomiting, stool, urine, and perhaps other emunctories, and that at the period when death arrives, whether as a consequence of the poisoning, or of other cause, not a portion of that poison is detected, which, had life been speedily destroyed, must have been infallibly discovered.

"The practitioner will then not conclude that poisoning has not occurred merely because he cannot detect the poisonous substance. He should be the more circumspect in this respect as his want of success, independently of the cause commented upon, may be owing to the imperfection of the procedures he has employed for its detection, or from its being one of those numerous substances which up to the present time elude our investigations. If it is true that we can by delicate analyses detect in the alimentary canal, the stools, or the vomits, notable portions of strychnia, brucia, morphia, prussic acid, &c., it is well known how difficult it is to demonstrate the presence of small portions of these substances when we have to search for them in the blood, or in organs to which they have been carried by process of absorption. We know, too, how powerless is art in the analysis of a number of active vegetable poisons, such as stramonium, henbane, aconite, hemlock, digitalis, &c., and that when the juices or extracts of these plants are mixed in a large proportion with the contents of the alimentary canal. In every case of poisoning, then, when the practitioner is unsuccessful in the detection of the substance employed, he ought, before pronouncing an opinion, to examine attentively all the circumstances which have preceded, accompanied, or followed the disease. Its nature and progress will in certain cases raise suspicions, or establish probabilities, and enable him to furnish an important element in the inquiry. In other cases, he must confine himself to declaring that it is not impossible that the patient has died poisoned, while sometimes he will be enabled to state that death is due to some other cause.

"In the second place, in many cases of poisoning, the practitioner *can only discover in the suspected matters an excessively small proportion of poison.* If there are cases in which none can be discovered, it is easily understood that there are others in which the most skilful chemists can only discover mere traces of its existence. Suppose that death, instead of occurring 10, 12, or 15 days after poisoning, takes place about the fourth or fifth day, it would be a strange error to state that the persons had not been poisoned, because only the few atoms not yet eliminated can be detected. It may be asked also what is the exact quantity necessary for the production of poisoning, and whether we are ever able to collect the whole of that which may be found in the body after death. All parts of the body cannot be operated upon, and the most skilful must lose a portion of the poison. Then again, the quantity obtained will much depend upon the skill of the operator. If this vague idea of the necessity of procuring a determinate quantity of poison prevails, numbers of guilty persons must escape justice to the great detriment of social order."

We may here express an opinion we have long entertained, that the machinery for the detection of poisoning stands in need of amendment in this country. Whether the investigation be conducted in a satisfactory manner is now a mere matter of chance. It rests with the members of that clumsiest of all medico-legal tribunals, the Coroner's Inquest, to determine whether a post-mortem examination and analysis shall or shall not be performed, and that these have often been neglected in order to save time for the jurors and fees for the county, is well known ; but, even supposing an analysis determined upon, what security is there that it shall be effectually performed ? The practitioner called in at hazard to attend the deceased may be, and in the majority of instances is, quite competent to the treatment of the case; but he will rarely be the person to whom the management of an analysis upon the effectual performance of which life and death may hinge should be entrusted. Manual dexterity, constant familiarity with the appearances of the various re-agents, and much other special knowledge, are here required, which a man may possibly possess shortly after leaving the schools, but which he cannot retain amid the multifarious and more frequently recurring duties of ordinary practice. This investigation should be consigned to certain special medical officers whose districts should be large enough to ensure a frequent recurrence of similar cases. The teachers of Medical Jurisprudence in our schools would form admirable officers for the investigation of this and other descriptions of sudden death: and indeed they are now voluntarily consulted by those practitioners who wisely decline taking upon themselves the responsibility of deciding upon appearances with which they are insufficiently familiar, and risking the compromising the lives and liberties of their fellow-creatures. Reports from officers of this description might be laid before the coroner's jury, or other more appropriate tribunal, as a foundation for future judicial procedure.

4. *Evidence from Experiments on Animals.*—M. Orfila has a long chapter upon this subject; and the conclusions he arrives at, based upon his extensive experience in this description of research, are interesting and important. He considers the effects which *poisons produce upon dogs to be identical with those observed in the human subject.*

"I can declare that, after having performed many thousand experiments upon dogs, and compared them with what is observed in man, that there is no difference in the nature of the symptoms and the organic lesions which poisons produce; and that the only difference exists in the extent of the dose required, in the influence of the *morale,* and in the relative strength of the animals—circumstances which can only influence the intensity of the symptoms and lesions, and consequently the duration of the disease. I shall support this assertion by a great number of facts, when I come to consider the individual poisons; at present I will refer only to a few of them. The caustic poisons, which especially cause death by producing a violent inflammation in the parts they touch and disorganize, should exert a similar effect upon all the animal tissues. And so experience proves that the concentrated acids and alkalis, the nitrate of silver, the protochloride of antimony, &c., produce in dogs a similar affection to that which they develop in man. Nux vomica, which powerfully excites the spinal marrow of dogs, acts upon man in the same manner; and indeed the idea of employing this substance in certain cases of paralysis first occurred to M. Fouquier, in consequence of Magendie and Delille's experiments upon dogs. It is true these animals are much more easily affected by nux vomica than is man, yet it is undeniable that the latter may die poisoned by it if he takes a sufficient quantity. If we examine attentively the effects which are produced on man and dogs by opium, prussic acid, hellebore, datura, belladonna, deleterious gases, &c. &c., we shall be obliged to allow that all these poisons produce identically the same effects on both species of animal."

But, to obtain exact results from these experiments, M. Orfila believes the *ligature of the œsophagus* indispensable; and he enters into a statement of considerable length to prove that the effects of this operation are not such as to confuse the observation of the results of the experiments. Careful experiments, he says, have assured him "that the conclusions I have drawn need undergo no modification on account of the ligature of the œsophagus; and that it is impossible to write a complete work on poisons without often performing this operation." He separates the œsophagus somewhat from its connexions, and having made a small opening into it, introduces the poison, and then ties a ligature below the aperture;—thus preventing the substance from accidentally entering the air-passages, and rendering its rejection by vomiting impossible. The effects of such ligature, when poison is *not* administered, are:—(1). It never produces during the *first two days* more than slight fever and depression, quite incapable of causing death in so brief a period. (2). If the animal is killed at this epoch, no lesion is discoverable. Thus, if an animal, to whom poison has been given, a short time prior to tying the œsophagus, dies within two days after suffering certain severe symptoms, we conclude it has died from the poison. So, too, with respect to the lesions found after death. "The influence which the ligature has exercised upon the animals I have poisoned may now be judged of—a number comprehending at least $\frac{7}{8}$ of those I have experimented upon, and which have died at 2, 4, 8, 12, or 24 hours after giving them the poison." (3). The fever and weakness consequent on the operation augment during the 3d, 4th, 5th days, and until death; and various other symptoms, such as vertigo, attempts to vomit, slight convulsions, manifest themselves, though frequently these are absent, and an utter insensibility is alone present. If the poison is one which acts very slowly, its effects and the lesions from the operation might be confounded. In such cases the experiment of producing the same effects without tying the œsophagus must be tried, or, if the œsophagus be tied without forming an opening in it, the animal is hardly inconvenienced for 36 hours. For the study of Poisons under their various relations *this operation is indispensable*. For, whether we investigate the action these substances exert upon the tissues, the symptoms they produce, or the antidotes they require, all is uncertain unless vomiting is rendered impossible; and the experiments of those authors who have not adopted this precaution, in which the animals have in consequence rejected a portion of the substance employed, are of no value whatever.

As to the value which should be attached to evidence derived from the symptoms induced in animals who have been compelled to swallow the contents of the stomach of persons supposed to have died poisoned, M. Orfila observes:—

"(1.) That experiments of this kind should never be tried if, by means of appropriate chemical agents, the observer is already enabled to demonstrate the presence of one or more poisonous substances. (2.) If the chemical inquiry has been fruitless, and there remains a portion of the suspected matter which has not been operated upon, it may be introduced into the stomach of a dog, and the effects observed. (3.) We should never employ for such experiment suspected matters which have already been submitted to the operation of re-agents, with a view to determine upon their poisonous nature—such re-agents being almost always themselves deleterious."

Conclusions can be satisfactorily drawn only when the symptoms following the introduction of the matters containing the suspected poison are acute and death prompt, and lesions corresponding to these are discovered after death. Even where death does result, it may sometimes do so from some disordered condition of the system of the individual having rendered his secretions, and especially the bile, deleterious. On the other hand, the animal not suffering does not prove poison has not been given. It may here become decomposed by the aliments or secretions of the stomach, or removed from among these by rejection in vomiting or absorption. At the best, all such experiments are but secondary and corroborative.

Dr. Christison comes to much the same conclusion of the value of these experiments in a medico-legal point of view.

"On the whole, it appears that, in the present state of our knowledge, experiments or accidental observations on the effects of the contents of the stomach, or of vomited matter, on animals, are equivocal in their import. At the same time it may be observed, as with regard to articles of food, drink, or medicine, that the effects of some poisons on man may be developed so characteristically on animals by the contents of the stomach, as to supply very pointed evidence indeed. Of the force of this statement the following example is a striking illustration. In the case of a girl, who was proved to have died of accidental poisoning by laudanum, the inspector evaporated the contents of the stomach to dryness, made an alcoholic extract from the residue, and giving this to several dogs, chickens, and frogs, found that they were all made lethargic by it, some of them oftener than once, and that a few died comatose. Facts, such as these, agreeing so pointedly with the known effects of the poison suspected, appear to me to yield evidence almost unimpeachable."

5. *Imbibition of Poisons.*—M. Orfila has an interesting chapter upon this subject. As the result of many experiments, he states that solutions of the salts of copper, injected into the stomach and rectum after death, penetrate by imbibition into even distant organs, in the course of several days, when the solution is sufficiently strong. The skin is traversed with great difficulty by such fluids.

"It is very difficult to admit that a corpse, the skin of which continues entire, will allow the easy passage of a poisonous liquid that may accidentally exist in the soil in which it is interred, because this liquid, absorbed in great part by the earth, would be very sparing in quantity, and at most able slightly to moisten it. At any rate, the subcutaneous cellular tissue, and still more the muscles and viscera, can only contain a small proportion of such poisonous fluid at the end of a very long space of time, if ever they contain it at all. It is true contrary results might be produced if the earth covering the corpse were watered for a long time daily with a poisonous fluid, or if the body could be left in a bath formed of a poisonous solution; but such a circumstance can never present itself in legal medicine without its being known, and it is therefore absurd to attach the slightest importance to it. In applying these conclusions to poisoning by *arsenic,* I must persist more than ever in an opinion I have delivered, viz. that the soil of a cemetery, even supposing it highly arsenical, which is never the case, will not yield arsenic to a corpse in such a manner as to lead to an opinion that poisoning has occurred. This I do in spite of the assertion of M. Devergie, for, independently of what I have said, the arsenical compound found in these soils is completely insoluble, even in boiling water."

From the whole enquiry two conclusions may be drawn—*first,* that all poisons, fluid or solid, providing they are not absolutely insoluble, may be, in cases of poisoning, carried into the various tissues, by imbibition after death, as well as by absorption during life, and which explains why the viscera furnish a larger portion, when examined several days after death than they do when examined during life, or very shortly after death; and *secondly,* that it is possible to procure from the viscera of animals, who have died of some other affection, a certain proportion of a poison which may have been introduced into the alimentary canal *after death.* This latter point is one of scientific interest, only, as M. Orfila states, the annals of crime in no country present an example of any wretch endeavouring to obtain the conviction of an innocent person by such diabolical means. He, however, enumerates the various modes of distinguishing the appearances caused by imbibition after death from those produced by poisoning; but for which we must refer the reader to the work itself.

6. *Moral Evidence.*—The various circumstances which usually constitute this, many of which the medical practitioner is often the person best qualified to take cognizance of, are thus enumerated by Dr. Christison:—(1) The suspicious conduct of the prisoner before the event, such as meddling with, or conversing concerning the properties of, poisons. (2) His having in his possession, or purchasing, poison upon false or insufficient pretences. Enough stress is not usually laid upon the fact of persons who excuse the possession of poison by their intention of destroying rats with it, not having warned the household of such circumstance. Neglect of this kind gives reasonable ground of great suspicion. (3) The administration of the poison either in food, drink or medicine. Some recent trials have broken down for want of proof of *administration.* Such proof can rarely be derived but from indirect sources, and the evidence of the medical man is often of great importance, he being generally the first person called, has the opportunity of securing any suspected articles of food for subsequent examination, and thus contributing to the proof of guilt or innocence. A case of poisoning is mentioned in which arsenic was detected in a parcel of oatmeal whereof the poisoned porridge had been made, and to which the prisoner had access, but was not found in the store whence the parcel was taken.

At other times the examination of the person of the accused leads to detection. A woman, having been poisoned by nitric acid, the stains of this substance were found upon the prisoner. Another important source of evidence is the observation of the period at which the symptoms commence after the administration of the substances supposed to contain poison. Thus, Dr. Christison gave an opinion that the death of a person poisoned by arsenic was not caused by a particular draught given by the accused eight hours before the symptoms commenced. Subsequent observation, however, has led him to the conclusion that sleep possesses the power of putting off for a while the action of some poisons. "In particular, some instances have occurred to me where arsenic, taken at night, did not begin to act for several hours, the individual having in the mean time been asleep." Where poisoning is effected by repeated moderate doses, this observation of the periods of occurrence of symptoms is very important, especially as chemical analysis may not in such cases be always satisfactory. In Miss Blandy's trial it was proved that the symptoms manifested themselves on each occasion she had administered the gruel to her father. The same correspondence was observed in the recent case of Laffarge. The special period at which symptoms manifest themselves may fix the guilt upon a particular person who might not otherwise have been suspected. Mrs. Humphreys, condemned for poisoning her husband with sulphuric acid, was proved to be the only person who could have obtained access to him just before the poison was poured into his mouth while asleep.

(4) The intent of the prisoner, such as the impossibility of his having administered the poison ignorantly, by accident, or for beneficial purposes. (5) The simultaneous illness of other members of the family. "It may be laid down as a general rule, that, perhaps if two, but certainly if three or more persons, after taking a suspected article of food or drink, are each affected with symptoms, furnishing of themselves presumptive evidence of poisoning, and have been seized nearly about the same time, and within the interval after eating within which poisons usually begin to act, the proof of poisoning is decisive. Several late cases might, in my opinion, have been decided by this rule." The escape of a portion of a party, while others of them suffer, is no proof of poison not having been administered. The poison may have been exclusively placed in the portions of food consumed by those who suffered; while, as Orfila observes, those who had filled their stomachs abundantly with food may suffer less than those who had eaten sparingly, especially if such repletion produce vomiting or alvine evacuations. A severity of symptoms proportionate to the quantities of poisoned substance consumed by different persons, is strong evidence.

(6) Suspicious conduct on the part of the accused during the illness of the person poisoned—"such as directly or indirectly preventing medical advice or relations being sent for, showing too much anxiety not to leave the patient alone with any other person, or attempting to remove or destroy articles of food or drink, or vomited matter, which may have contained the poison, or expressing a foreknowledge of the probability of speedy death." Dr. Christison thus states the duty of the practitioner.

"In such a conjuncture he is undoubtedly placed in a situation of some delicacy. But, on considering the matter attentively, good reasons will appear why he should adopt the course, which, I believe our courts of justice will expect of him, and keep some watch over the actions of any individual who is suspected of having committed the crime. On the one hand, no one else is, by education and opportunities, so capable of remarking the motions of the different members of the family dispassionately, without officiousness, and without being observed. And, on the other hand, it is undoubtedly a part of his private duty as practitioner, to protect his patient against any farther criminal attempts, as well as part of his public duty to prevent the vomited matter and other subjects of analysis from being secretly put away or destroyed. No one can be so occupied without many accessary particulars coming under his notice. And certain it is, that on several trials the practitioner has contributed, with great credit to himself, a considerable part of the pure moral proof. For an example of discreet and able conduct under these trying circumstances, the reader will do well to refer to that of Dr. Addington, the chief crown-witness, both as to medical and moral facts, in the case of Miss Blandy. It is almost unnecessary to add that, in acting as now recommended, the physician must conduct himself with circumspection, in order to avoid giving unnecessary offence, or alarming the guilty person."

(7) Suspicious conduct after the person's death, such as hastening the funeral, preventing or impeding the inspection of the body, giving a false account of the previous illness, showing an acquaintance with the real or supposed effects of poison upon the dead body. (8) The personal circumstances and state of mind of the deceased, his death-bed declaration, and other particulars, especially such as tend to prove the impossibility or improbability of suicide. Declarations of the sufferer made to the medical attendant, may be of the most vital import, and cannot be too carefully taken. Dr. Christison particularly dwells upon the importance of this rule in reference to the history of the symptoms, prior to the practitioner being called in.

" On this part of the history, including particularly the time and manner in which the illness began, medical conclusions of extreme consequence are often subsequently founded. On a single fact or two may depend the fate of the prisoner. It is not enough, therefore, in my opinion, that such evidence formed part of the death-bed declaration. If a fact derived at second-hand from the deceased, and stated too by him from memory, is a material element of any of the medical opinions on the trial, it is of much importance that the information be procured by a medical man: and that the person who procured it, whether professional or not, was aware at the time of the probability of its becoming important. Such evidence, although not collected with these precautions is admissible; but I have so often had occasion to witness the carelessness with which the previous history of cases is inquired into, both in medical and in medico-legal practice, that I do not see how it is possible to put trust in evidence of the kind, unless it bears marks of having been collected with care, and under an impression of its probable consequence."

This section upon the moral or circumstantial evidence is very ably written, and should be diligently perused by every medical man; for, notwithstanding the complimentary admission by our author of the excellent nature of the medical evidence upon some trials, its defective character in others is notorious, and that when witnesses of high repute have been under examination too.

We now pass on to notice some of the individual poisons, of which

### ARSENIC

First claims attention, both from the frequency with which it is employed as the agent of destruction, and from the additional information respecting it, supplied in the two treatises under review. Of 543 fatal cases of poisoning upon which Coroner's Inquests were held in England during two years, death was caused in 186 by arsenic; in 35 by mineral acids; in 19 by oxalic acid; in 15 by corrosive sublimate and other mercurial preparations; in 9 by various mineral irritants, as tartar emetic, sulphate of iron, &c.; in 9 by vegetable irritants, as colchicum, savine, &c.; in 2 by lytta; in 193 by preparations of opium; in 34 by prussic acid; in 18 by various vegetable narcotics, as nux vomica, strychnia, &c.; in 2 by coal gas; and in 22 by poisons whose nature was not declared. Of 288 cases of poisoning in France, 196 were caused by arsenical compounds.

1. *Tests for Arsenic* (*Arsenious Acid*).—It shows the facility with which difference of opinion may be entertained, even in cases apparently admitting of easy decision, that Dr. Christison and M. Orfila are at issue with respect to the *taste* produced by arsenic. The latter states it to be "rough but not corrosive, slightly styptic, only perceptible at the end of a few seconds, persistent for a long time, and exciting salivation in a marked degree. Dr. Christison is wrong therefore in stating it to be insipid." Dr. Christison retorts upon the Professor.

"The reader will find some details upon this point in a paper I published in the Edinburgh Journal (vol. xxvii.) In the present work it is sufficient to observe, that I have repeatedly made the trial, and seen it made at my request by several scientific friends; and that, after continuing the experiment as long, and extending the poison along the tongue as far back, as we thought safe, we all agreed that it had scarcely any taste at all—perhaps towards the close a very faint sweetish taste. It appears to me that the experiments made on that occasion, might have set at rest the question as to the taste of arsenic, and corrected an important error long committed by systematic authors in chemistry as well as medical jurisprudence. And accordingly in this country, the truth is now generally known. Professor Orfila, however, continues to repeat the error. * * * * * * * * It is impossible to make satisfactory experiments with safety on its impressions at the back of the palate. But we may rest assured that in general it makes no impression there at all; for it has been often swallowed unknowingly with articles of food. Not a few have in such circumstances noticed merely its grittiness, and thought there was sand in their food. Two instances only am I hitherto acquainted with, where an acrid sensation would seem really to have been experienced in the act of eating or swallowing. It is not improbable that, in an *ex post facto* description, the reporters, as others in the same circumstances have clearly done, confounded the subsequent inflammation with mere taste in the act of chewing or swallowing."

It is not our intention to reproduce here the details of the various tests which are exhibited in all their minutiæ in the works of Orfila and Christison, works which, for all practical purposes of analysis, any abstract would not serve as a substitute for consulting. Arsenic in its *solid* state may be most satisfactorily detected by reduction and subsequent oxidation, by which means a 300th part of a grain can be discovered. The alliaceous odour and other supplementary tests are not deserving of retention. Existing in the state of *solution*, this poison may be detected by the *liquid tests*, Hydrosulphuric Acid, Ammoniacal Nitrate of Silver, and Ammoniacal

Sulphate of Copper. Dr. Christison thinks these have been too much neglected of late; for, although any one used alone may be open to fallacies, their conjoint indications are quite satisfactory. By the generation of hydrogen in an arsenical mixture with *Marsh's apparatus*, and subsequently decomposing the arseniuretted hydrogen by combustion, a millionth part of oxide may be detected. The identification of the metallic crust produced by this process has given rise to a great variety of supplementary tests. The process of *Reinsch* by which $\frac{1}{250000}$ gr. may be detected, consisting in precipitating the arsenic on copper, is the simplest and easiest of all, and liable to no important fallacy. In the great majority of instances arsenic has to be detected in *organic mixtures*, such as the contents of the stomach, vomits, &c. Vast additional interest attaches to the subject in the wide field opened for research by the discovery by Orfila in 1839, (and since repeatedly acted upon both in France and in this country) of the possibility of detecting this substance within the intimate texture of various organs to which it has been conveyed by absorption. A large portion of the first volume of the "Toxicologie" is occupied in detailing the varieties in the process required for the detection of this poison in its various organic combinations. The method employed in general, consists in the destruction of the animal matters by the agency of nitrate of potash, nitric acid, or sulphuric acid, and submitting the resulting solution to a modification of Marsh's apparatus.

Various *objections*, however, have been offered to the conclusion that the recovering of arsenic from the intimate textures of the frame, is to be considered a proof of the poison having been administered during life: these have been answered at some length by Professor Orfila. 1. *Arsenic* it is said may *exist in the re-agents employed.* This is the case sometimes with sulphuric and muriatic acids, and is said to be so occasionally with nitric acid, nitrate potass, and even zinc. The submitting all materials to be employed to a preliminary examination affords an easy mode of obviating this source of fallacy. 2. *It may be present in some articles of chemical apparatus.* Thus, the iron of the pots recommended by Orfila to be employed, when the analysis is conducted on a *large scale*, contain a little arsenic; but this is not separated from the iron during the processes employed. Both the objection and reply to it, as Dr. Christison observes, are now of comparatively slight importance, for, since the discovery that certain organs and excretions, as the liver and urine, are especially the depositaries of the poison, the submitting of the whole carcase to analysis can never be necessary; and the public will be spared the repetition of the *melodramatic* scenes which we think disgraced the tribunals of justice in a neighbouring country during some recent celebrated trials. Dr. Christison may well say, no British Jury would pronounce a prisoner guilty "when the analyst, after boiling an entire body, with many gallons of water, in a large iron cauldron, making use of whole pounds of sulphuric and nitric acids, and nitre, and toiling for days and weeks at the process, could do no more than produce minute traces of the poison. What man of common sense will believe that, with such bulky materials and crude apparatus, it is possible to guard against the accidental admission of a little arsenic." 3. *Arsenic may have existed in the antidote used.*—Thus, a little arsenic may now and then exist in the iron whence the hydrated sesqui-oxide is prepared. Portions of the stock whence it was taken should be therefore examined. 4. *It may exist naturally in the human body.*—Orfila at one time felt certain he had detected it in the bones, but has never been able to repeat the demonstration. Its normal existence within the bones and in the soft parts has been frequently urged in the French law-courts; but he and most other toxicologists now believe without any foundation in fact. 5. Arsenic has been found by M. Orfila in some *cemeteries,* but as an arsenite insoluble in even boiling water, so that it cannot be believed that this could penetrate into the textures of the body, even when the coffin has been destroyed by accident or time. Indeed, experiment shows how difficult it is to cause even a soluble arsenite to traverse the tissues, when the body continues entire. When this occurs, organs will be found penetrated according to their bulk and proximity, while arsenic resulting from poisoning is found to bear some proportion to the vascularity of the organs it is found in. 6. *The arsenic may have been taken medicinally.*—Here almost everything depends upon the history of the case, as regards symptoms, dose of the arsenic, period of taking it, &c. M. Orfila offers many diagnostic observations upon this point; but we must refer the reader to his work for them.

2. *Of the Action of Arsenic, and the Symptoms it excites in Man.*—Dr. Christison's chapter upon this division of the subject is a most able one, but we can only present a brief outline of its contents. The operation of arsenic by entering into the blood, before rendered so probable, has been indisputably proved by the discoveries of Orfila. Its action upon this fluid is not well ascertained, beyond the greater degree of fluidity it causes in most cases of poisoning. The heart is the organ remotely acted upon in cases of rapid death following the taking of the poison, while, where life is more prolonged, the general nervous system suffers. It acts with nearly the same energy, whatever texture it is applied to, and death has followed its application to wounds or ulcers, the abraded cuticle, the lining membrane of the rectum, vagina, and air-tubes. So small a quantity as two grains of the white oxide has been said to have caused death, and less quantities produce various alarming symptoms. The action of even large doses much depends upon the state of repletion of the stomach, being far most vigorous when this organ is empty. Dr. Christison divides the cases of poisoning by arsenic into three classes, each manifesting some difference in the nature or course of the symptoms.

1. In the first, and by far the most numerous, class of cases, there is "violent irritation of the alimentary canal, and sometimes of the other mucous membranes also, accompanied by excessive general depression, but not with distinct disorder of the nervous system." The person usually survives the 24 hours, but seldom more than three days. In some cases he dies in a very few hours, or lives for weeks. Sickness and faintness usually come on in about half an hour, though sometimes long before, while, in a few cases, especially where sleep has occurred, they have been delayed for two, three, five, or eight hours. We need not follow the train of symptoms here graphically set forth. It will suffice to say that, after those produced by irritation of the alimentary canal have continued a few hours, convulsions and cramps supervene, and the pulse, which was feeble at the commencement, becomes imperceptible. Delirium or stupor often accompany the advanced stage; and, after the horrid agonies of the early stage of the affection, death is frequently calm enough. The symptoms in these cases are usually uniform, but in some rare cases, even pain and vomiting have been entirely wanting.

2. In the second set of cases there is little sign of irritation of the alimentary canal—excessive prostration with fainting being the principal feature, and death occurs in five or six hours, before inflammation can have developed itself. In a few cases the symptoms have approached to narcotism, as they did in some of Brodie's experiments. This variety of poisoning has been observed only when the dose has been very large, when it was in little masses, or when it was in solution. These cases are not common, but should command the attention of medical jurists, as an erroneous opinion still too generally prevails, that poisoning by arsenic *always* produces violent and well-marked symptoms.

3. In this set of cases there are two distinct stages in the progress of symptoms, first the signs of inflammatory action, ordinarily violent, of the alimentary canal, followed, on the second, fourth day, or later, by those of affections of the nervous system, *e. g.* palsy or epilepsy. These are usually cases in which a small quantity has been taken, or prompt vomiting is induced. Usually, recovery eventually takes place, although death may follow a protracted illness. The nervous affection may show itself during the existence of the inflammatory action.

We extract some of Dr. Christison's valuable remarks upon the strength of evidence supplied by symptoms in arsenical poisoning.

"The present doctrine of toxicologists and medical jurists seems generally to be, that symptoms alone can never supply decisive proof of the administration of arsenic. This opinion is certainly quite correct when applied to what may be called a common case of poisoning with arsenic, the symptoms of which are little else than burning pain in the stomach and bowels, vomiting and purging, feeble circulation, excessive debility, and speedy death. All these symptoms may be caused by natural disease, more particularly by cholera; and consequently every sound medical jurist will join in condemning unreservedly the practice which prevailed last century of deciding questions of poisoning in such circumstances from symptoms alone. But modern authors appear to have overstepped the mark, when they hold that the rule against deciding from symptoms does not admit of any exceptions. For there are cases of poisoning with arsenic, not numerous certainly, yet not very uncommon neither, which can hardly be confounded with natural disease; and, what is of some consequence, they are precisely those in which the power of deciding from symptoms alone is most required, because chemical evidence is almost always wanting. Either the peculiar combination of the symptoms is such as cannot arise from natural causes, so far at least as physicians are acquainted with them; or these symptoms occur under collateral circumstances, which put natural causes almost or altogether out of the question."

In illustration of this opinion, Dr. Christison refers to some cases in which affection of the whole of the mucous membrane followed the taking certain substances; and others, in which distinct nervous affections followed the inflammatory one. That such should arise from ordinary disease is hardly possible, unless the cause be also known.

"But to conclude, there are likewise collateral circumstances connected with the symptoms, which, taken along with the symptoms themselves, will sometimes place the fact of poisoning with arsenic beyond the reach of a doubt. Thus, if a person were taken several times ill with symptoms of general inflammation of the mucous membranes, after partaking each time of a suspected article of food or drink, the proof of the administration of arsenic would be very strong indeed; and it would be unimpeachable if at length a nervous affection succeeded at the usual period. Or above all, suppose several persons, who have partaken of the same dish, are seized about the same time with nearly the same symptoms of irritation of the mucous membrane. The proof of general poisoning would then be unequivocal. And if one or more of them should afterwards suffer from a nervous disorder, little hesitation ought to be felt in declaring that arsenic is the only poison which would have caused their complaints."

The case of Miss Blandy may be cited as an example of the first, and that of Eliza Fenning of the last, of these statements.

3. *Morbid Appearances.*—We regret we cannot pursue the interesting detail of these, given by Dr. Christison; but we have only space to extract his opinion of their juridicial value.

"On reviewing what has been said of the pathological appearances caused by arsenic, it must appear that the medical jurist can never be supplied from this source alone with satisfactory evidence of the cause of death. But, in some circumstances, the evidence may amount to a strong probability of one variety or another of irritant poisoning. Mere redness, conjoined or not with softening of the mucous membrane, may justify suspicion only. But if there should be found in the body of a person who has died of a few days' illness, redness, black warty extravasation, and circumscribed ulcers of the villous coat of the stomach,—effusion of blood or bloody clots among the contents of that organ,—also redness of the intestines, more especially redness and ulceration of the colon and rectum,—and redness of the pharynx, or of this along with the gullet,—the proof of poisoning with some irritant will amount to a strong presumption. At least, it is difficult to mention any natural disease which could produce in so short a time such a conjunction of appearances as this; which arsenic and other analogous poisons sometimes occasion."

There are some good remarks upon the remarkable preservation from decay poisoning by arsenic confers upon the corpse. This might be made a corroborative proof; and is at all events a fortunate circumstance, for the poison may be detected in the undecomposed body months or years after death.

We may subjoin a caution, delivered by Professor Orfila, as to one morbid appearance, which has sometimes been a source of error. He says:

"I cannot quit this subject without observing that, under certain circumstances, there is observed, here and there, in the stomach and intestines of persons poisoned by arsenic, a number of brilliant points, which one would at first be tempted to believe to be arsenious acid. These grain-like bodies are formed of fat and albumen. Placed on live coals, they decrepitate with a noise, which has improperly been termed detonation. They inflame when they contain a large proportion of fat, and give out an odour of burning animal matters. They are found also in the bodies of persons who have not been poisoned. Billard has seen them in two women: one of whom, aged 72, died of chronic gastro-colitis; and the other, aged 50, of phthisis, having many ulcers in her intestines."

4. *Treatment of Poisoning with Arsenic.*—Several substances have been proposed from time to time as antidotes for arsenic, such as sugar, lime-water, oils, liver of sulphur, or sulphuret of potassium, &c.; and M. Orfila has exposed at large their inefficacy. Magnesia, or charcoal, given in large quantities shortly after the poison has been swallowed, seems sometimes to have been of use, by covering the particles of the arsenic with its insoluble powder. But the only substance which has any pretension to the character of *an antidote,* is the *hydrated sesquioxide of iron,* which, brought in contact with oxide of arsenic, forms an arsenite insoluble in the secretions of the stomach. Some difference of opinion as to the beneficial effects of this substance has been entertained among observers. But when it has been resorted to sufficiently early, its inefficacy would seem often to have arisen from insufficient doses having been employed. To remove one part of arsenic from a state of solution, twelve parts of oxide in its moist state, and sixty parts if previously dried, are required. Orfila states that the resulting arsenite is somewhat soluble in the secretions of the stomach, and is poisonous to animals; but that this effect may be counteracted by giving the oxide in excess, so that the compound may be thrown down again as fast as dissolved. This antidote has been found repeatedly successful, and should be kept ready prepared by every druggist.

An antidote, however, can seldom be had recourse to early enough, and if spontaneous and free vomiting do not occur, it must be excited by emetics, administering at the same time milk in considerable quantities for the purpose of enveloping the poisonous matter. Emetics are not required if natural vomiting exists; and the stomach-pump possesses no advantage over either. When the poison has been evacuated, we have the inflammatory state of the alimentary canal to deal with; and the active antiphlogistic measures indicated for its relief may be injurious in the condition of great prostration which usually prevails. Still, active venesection seems to have saved several cases. Orfila recommends it as a means of removing a portion of the poison circulating with the blood; but forbids it prior to the due evacuation of the stomach, as absorption would be increased, as also when all degree of re-action is absent. Rasori and Rognetta recommend the stimulant mode of treatment; Orfila, as the result of 157 experiments on dogs, declares such treatment as useless, and even prejudicial. "At the same time," as Dr. Christison observes, "no one who has ever seen a case of poisoning by arsenic, can doubt that it is often necessary to counteract the overwhelming languor of the circulation by the moderate use of stimulants."

Dr. Christison suggests that, where the poison has been all evacuated, and the patient's strength admits of it, copious depletion, followed by full *opiates,* would form proper treatment, just as in ordinary inflammation.

Professor Orfila lays great stress upon the free employment, *after the active evacuation of the poison,* of a *diuretic potion,* composed of 10 pounds of water, 5 of French white wine, a bottle of Selzer, and 3 ozs. of nitre, the dose being two wineglassfuls frequently. He says,

"If this were taken abundantly during the early stages of poisoning, it would have the serious inconvenience of dissolving the arsenious acid and favour its absorption. The utility of this plan cannot be contested after the numerous experiments I have tried. It may be seen in my memoir in the Archives Generales,

Sept. 1841, that all the animals poisoned by the external application of arsenic, who would have died had they been left to themselves, recovered in a short space of time, after I had succeeded in obtaining an abundant passage of urine from them: and it will be observed that the urine, especially that passed during the first few days, contained notable portions of arsenic. Here experience confirms what theory would predict; for, in expelling the arsenic through the urinary passages, which is ready to destroy the vitality of organs, one acts as surely, as when we seek to remove it from the alimentary canal by vomiting and purging."

M. Agouard relates a case in the human subject successfully treated on this principle.

### Oxalic Acid.

It is somewhat surprising, considering the far greater rapidity and certainty of its action, that this substance is not much more frequently employed than arsenic for the purpose of suicide or murder. In the great majority of cases it has been taken inadvertently for sulphate of magnesia. Such accidents cannot be guarded against by attention to the different physical appearances of the two substances, for Dr. Christison observes, "that repeatedly, on desiring several persons to point out which was the poison and which the laxative, I have found as many fix on the wrong as on the right parcel." The acid taste and inferior solubility of the poison are safer guides.

Oxalic acid acts very differently, accordingly as it is employed in a concentrated or diluted condition. Given to dogs in the former state, it causes dreadful pain and effort to vomit, and if the dose is large, produces death in from two to twenty minutes. The stomach is found to contain black extravasated blood, the surface of its inner coat being brittle in places, having the subjacent stratum gelatinized. Diluted with twenty parts of water, the acid exerts scarcely any irritating effects upon the stomach, death, however, no less certainly resulting, the heart, spine, and brain being the organs affected. It produces nearly the same effects to whatever texture it is applied. It has never been detected in the blood; and although Orfila detected it in the urine, he has never found it in the liver and spleen.

In man, recovery from this poison is rare, where the dose is considerable, and that even in cases where abundant vomiting comes on at once. Death has occurred as soon as ten minntes, and few who take large doses survive more than an hour, although some remarkable exceptions are on record. Among fatal cases, the smallest dose hitherto taken has been half an ounce. Although signs of violent irritation of the alimentary canal are usually discovered after death, in some cases none whatever have been observed.

Owing to the rapid action of this poison, *treatment* is too often out of the question. The administration of an antidote which produces with the acid an insoluble compound, as chalk or magnesia, or in default of either, plaster from the wall is indicated. Alkalis, forming soluble oxalates are inadmissible. When the former are at hand, time must not be lost in exciting vomiting, or in using the stomach-pump, which "fashion seems to have authorised the employment of for every kind of poison ;" and care must be taken not to increase the solubility of the poison by encouraging vomiting by the ingestion of large quantities of fluid. Poisoning by oxalic acid, Dr. Christison states, may be inferred from symptoms alone.

"If a person, immediately after swallowing a solution of a crystalline salt, which tasted purely and strongly acid, is attacked with burning in the throat, then with burning in the stomach, vomiting, particularly of bloody matter, imperceptible pulse and excessive languor, and dies in half an hour, or still more in 20, 15 or 10 minutes, I do not know any fallacy which can interfere with the conclusion, that oxalic acid was the cause of death. No parallel disease begins so abruptly and terminates so soon; and no other crystalline poison has the same effects."

Professor Orfila enters at considerable length into the examination of the tests for the presence of this substance, and the fallacies which the existence of binoxalate of potash in sorrel soup, so much used in France, may give rise to. Rhubarb-root, too, contains always some, and sometimes a large proportion, of oxalate of lime.

### CORROSIVE SUBLIMATE.

All the circumstances relating to this and other mercurial poisons are very elaborately developed by both Dr. Christison and M. Orfila ; but we can only allude to a few of them. Before the discoveries consequent upon Orfila's improved modes of analyses, it had been long believed that this and other mercurial preparations acted through the medium of the blood. Many instances, some very hypothetical, have been narrated of live quicksilver being discharged from the body during a mercurial course ; and others, where this fluid was found in the bones after death. Buchner and Schubarth stated that they had obtained mercury from the blood and other fluids ; but competent observers have not been able to do so in employing these processes. Orfila, though unable to detect it in the blood, has repeatedly found it in the urine and liver of animals poisoned by, or of patients taking, the sublimate. The corrosive sublimate, when swallowed in poisonous doses, produces violent corrosion of the stomach, " and, however introduced, causes irritation of it and the rectum, inflammation of the lungs, depressed action, and perhaps inflammation of the heart, oppression of the functions of the brain, inflammation of the salivary glands." Frequently resembling *arsenic* in its corrosive action, it differs from it, inasmuch as the taste it causes, and the irritation and acridity it produces in the act of swallowing are instant and far more marked, so as sometimes to have led to a timely desisting. The countenance is not ghastly, but flushed ; and the discharge of blood by vomiting and purging is more frequent. The urinary passages are more irritated, and the nervous symptoms are more apt to come on during the inflammatory stage. Recovery after even large doses is more frequent, and deviations from the ordinary course of symptoms more rare.

Dr. Christison believes that symptoms alone will sometimes supply sufficient evidence of poisoning by sublimate.

"If after something has been taken which tasted acrid, and caused an immediate sense of heat, pricking, or tightness in the throat, the characteristic signs of poisoning with the irritants (especially if these are attended with the discharge of blood upwards and downwards) make their appearance in the usual time, and are soon after accompanied or followed by true mercurial salivation,—it may be safely inferred that some soluble compound of mercury has been taken. Before drawing this inference, however, it will be necessary to determine with precision all the classes of symptoms, more particularly the nature of the salivation. It should also be remembered, that salivation may accompany or follow the symptoms of inflammation in the stomach, in consequence of calomel having been used as a remedy. But if proper attention be paid to the fallacies in the way of judgment, I conceive that an opinion on the question of poisoning with corrosive sublimate may be sometimes rested on the symptoms alone."

*Treatment.*—M. Orfila, having proved the inefficacy of the various antidotes formerly in use, was fortunately enabled some years since to discover a very efficacious one in an accessible substance, namely, the *albumen* contained in whites (and yelks) of eggs. This has saved several lives. He observes: 1. The precipitate of albumen and the sublimate may be taken in a large dose without danger. 2. It is poisonous (but less so than the sublimate) when dissolved in excess of albumen. 3. Therefore, animals to whom more albumen than sufficient to produce the precipitate, is given, die, if they are prevented vomiting. 4. Dogs poisoned with sublimate, to whom excess of albumen is given, in consequence of the vomiting which the combination produces, survive. 5. Animals to which an insufficient quantity of albumen has been given, die in three or four hours. 6. Although albumen does not completely neutralize the poison, it is by far the best antidote discovered, because it is harmless in itself, does not form a hurtful compound unless in excess, and because, being accessible to all, it can at once be swallowed off. Dr. Wright says, that if its administration be followed up by an astringent decoction, or infusion, the solubility of the compound in excess of albumen is diminished. In a case of poisoning, eggs copiously diluted with water, or in a gum emulsion, should be given, and the stomach distended with fluid, so as to induce vomiting. If eggs are not at hand, rice-water, flour and water, sugar and water, or milk may be used. Vomiting is better excited in these cases by *abundant drinks*, which allay irritation, than by emetics. They should be therefore even forced upon the patient. If the taking the poison be followed by inflammation of the alimentary canal or peritoneum, general and local bleeding, fomentations, and anodyne or emollient glysters will be required. Edwards and Dumas state they have found iron filings an efficacious antidote in their experiments. In a supplement, Orfila notices the *protosulphuret of iron*, recommended as a good antidote by M. Mialhe; and declares 1. That it quite destroys the poisonous properties of the sublimate, if taken in a sufficient dose immediately. 2. It is inefficacious if not given within ten minutes. 3. Although a more complete antidote than albumen, if given instantly, in almost every case it could not be procured until the latter had already began to produce its beneficial effects.

### Lead.

We shall confine ourselves here to a brief abstract of Dr. Christison's researches upon the action which water and acids, under different circumstances, exerts upon metallic lead.

1. *The Action of Air and Pure Water.*—Lead exposed to the air, especially when moist, becomes tarnished by the deposit of a thin layer of carbonate of lead. Distilled water, deprived of air by ebullition, has no action on lead. If the water contain its usual gases, the surface of the metal becomes white and dull, and if it continues to have access to the air, copious white matter floats on the water or falls to the bottom. This, a carbonate of lead, forms with great rapidity. In twenty months 120 grains have been obtained from one ounce of lead kept in 24 ounces of distilled water. During all this, a minute quantity of lead is dissolved. Distilled water, therefore, for economical use, should not be kept in contact with lead.

2. *Action of Solutions of Neutral Salts.*—These impair the corrosive power of water, enabling us to employ lead for various economical purposes. The degree of preservative power varies much in different salts. Arseniate soda possesses it perfectly in the proportion of a 12,000th part. Phosphate of soda and hydriodate of potash are nearly effectual in the proportion of a 30,000th. Acetate of soda is only imperfectly so in 100th. Muriate of soda is a preservative in 2000th part, and sulphate lime in a 4000th. Lead, exposed for a few weeks to a solution of the protecting salt, acquires a film, after which even distilled water possesses no power over it. The protecting power depends upon the acid, not on the base of the salt, those salts possessing most preservative power, whose acids form with the lead insoluble salts. No lead can ever be detected in even weak preservative solutions.

"The general result of these experiments appears to be, that neutral salts in various, and for the most part minute, proportions, retard or prevent the corrosive action of water on lead—allowing the carbonate to deposit itself slowly, and to adhere with such firmness to the lead as not to be afterwards removeable by moderate agitation—adding subsequently to this crust other salts of lead, the acids of which are derived from the neutral salts in solution—and thus at length forming a permanent skreen, through which the action of the water cannot any longer be carried on."

3. *Action of Natural Waters on Lead.*—Lambe stated erroneously that *Rain* or *Snow Water* does not corrode lead. Collected in its pure state in the country, it acts nearly as powerfully upon any leaden gutter or receptacle as distilled water would do; and even in towns, where the presence of some preservative salt may cause a protective incrustation of carbonate of lead, this may become dissolved by the water, if any acid emanations, from manufactories, &c. exist in the neighbourhood. From the presence of muriates and sulphates, *Spring Waters* usually exert little or no action upon lead, although many examples are upon record in which, owing to the salts they contained being but of a very feebly protecting character, colic and other symptoms of lead-poisoning have resulted from their employment; and Dr. Lambe, amidst many valuable observations, erroneously comes to the conclusion, that all spring water acts thus prejudicially upon lead. This action of the water upon lead often occurs when it is kept constantly in contact with it, as in cisterns, (manifesting its action especially on the lids of these when of lead,) perhaps for months, while by merely passing over the metal it would have produced no effect upon it. Too pure spring water may thus often have this property of acting upon lead corrected by allowing it to remain in contact with the leaden receptacles for some months before it is drawn off, so that it may deposit a protecting crust of carbonate; or by filling the cisterns, &c. during the same period, with a solution containing $\frac{1}{25000}$ phosphate of soda. All risk is avoided by a recent patent process for lining and covering pipes with a thin coating of tin.

4. *Action of Acidulous Fluids on Lead and its Oxide.*—The effect of *carbonic* acid is not well ascertained. Water feebly acidulated with *sulphuric* acid acts much less rapidly on lead than when pure, while *hydrochloric* acid slightly favours the solvent power. *Acetic* acid, even when much diluted, dissolves metallic lead, provided its oxidation be effected by due exposure to the air. *Citric* acts more slowly, and *Tartaric* still more so. These vegetable acids act much more vigorously on the *Protoxide of Lead,* while the presence of air is not then required. In consequence of this action of the vegetable acids, articles of food are often rendered poisonous, as *e. g.* milk, wine, cyder, when brought in contact with it. Lead is now abandoned in the manufacture of cyder-apparatus in this country; but the adulteration of this drink in France with lead gave rise very recently to the production of colic. The metal is also much used there for the correction of the acidity of inferior wines.

### Hydrocyanic Acid.

The great increase of cases of poisoning by this substance of late invests it with great importance. The *tests* of its presence are its odour, the salts of copper, salts of iron, and nitrate of silver. The *odour* is a very delicate test, and, according to Orfila, is perceptible when no re-agent is delicate enough to detect it. Dr. Christison doubts this, and has "known some persons insensible of any smell, even in a specimen which was tolerably strong. Hence, when the odour is resorted to as a test, it ought to be tried by several." *Sulphate of Copper* and a little potash form a greenish precipitate with prussic acid, which becomes nearly white on the addition of hydrochloric acid. Lassaigne states this will act on poison dissolved in 20,000 parts of water. This is a far inferior test to the salts of the *mixed peroxide and protoxide of Iron,* which, on the addition of a little sulphuric acid, give a deep Prussian blue colour. Common green vitriol answers very well. *Nitrate of Silver* is a very delicate test—the cyanide of silver being insoluble in any but boiling nitric acid, by which it is decomposed, forming nitrate of silver, and giving out prussic acid. Or, if the precipitate be dried and heated, it gives out cyanogen gas, known by its beautiful rose-coloured flame. MM. Leuret and Lassaigne and Orfila have each devised processes for detecting prussic acid in mixed fluids. Orfila protests against the conclusions which have sometimes been drawn as to the presence of this substance without the production of cyanogen gas, leaving the case as imperfect as if in testing for poisoning by arsenic the metal was not produced. He also demands whether the detection of the poison in the vomits, alimentary canal, or liver of a person supposed to have died poisoned, is sufficient to prove poisoning to have occurred. He replies it is not, for—1. It has been occasionally known to be developed in some of the secretions in persons in a state of health or even a diseased condition. 2. It has not been proved that this acid may not be generated during the process of putrefaction. He admits this last observation is of little practical value.

*Action and Symptoms.*—Dr. Christison has endeavoured, in repeating Magendie's experiments with concentrated acid, to mark the periods of the commencing operation of the poison, and when death occurs. He says—

> "I remarked that a single drop, weighing scarce $\frac{1}{3}$ gr. dropped into the mouth of a rabbit, killed it in 83 seconds, and began to act in 63 seconds—that 3 drops weighing $\frac{4}{5}$ gr. in like manner killed a strong cat in 30 seconds and began to act in 10,—that another was affected by the same dose in 5 and died in 40 seconds, —that 4 drops weighing 1 gr. $\frac{1}{5}$ did not affect a rabbit for 20 seconds, but killed it in 10 more,—and that 25 grs. corresponding with $1\frac{1}{2}$ oz. medicinal acid, began to act on a rabbit as soon as it was poured into its mouth, and killed it outright in 10 seconds at farthest."

Magendie has killed animals apparently instantly, and Dr. Thomson in 2 seconds. Mr. Blake declares all experiments representing the action of the poison to have begun before 10 seconds are fallacious, as he could never succeed in inducing action in less time even by injecting 30 minims into the femoral veins. But, as Dr. Christison well remarks, negative results cannot outweigh careful positive observation. In the rapid cases death took place just as tetanic symptoms, which prevail in slower cases, were commencing. Diluted acid produces the same effect when the dose is large enough. When it is smaller the animal may suffer from giddiness, insensibility, or convulsions from half an hour to a whole day. The acid acts on the brain and on the spine, producing the combination of coma and tetanus. There are few morbid appearances beyond turgidity of the cerebral vessels, and a distension of the heart and large vessels by black blood. There is difference in the statements of different observers, as to whether the irritability of the heart is exhausted or not. All animals, high or low in the scale of the creation, are destroyed by it, and much in the same manner. It is not believed to be a cumulative poison. In man, large doses extinguish life in a few seconds, or at all events in a few minutes; but if the individual survives 40 minutes he usually recovers. The fixing the period when the poison begins to act, is often a very important point in medico-legal investigations; and some very recent cases, observed by Dr. Letheby and others, would seem to show that more actions may be performed after taking large doses prior to the occurrence of death than was heretofore believed possible. In some of these, however, the exact quantity taken is a mere matter of uncertain inference and conjecture. The fixing the smallest fatal dose is important. Seven epileptics were killed in a Parisian hospital by the administration of $\frac{2}{3}$ gr. of pure acid to each, and as several did not die for 45 minutes, a less dose would probably not be fatal.

The peculiar appearance of the eye some have laid so much stress upon is thus commented upon.

"It appears that even long after death the eye, as in Hufeland's case, has a peculiar and staring expression, so as to render it difficult to believe that the individual is really dead: and this has been considered by Dr. Paris as so remarkable, as even alone to supply 'decisive evidence of poisoning by hydrocyanic acid.' But the accuracy of his opinion may be questioned. The appearance is indeed very general in cases of poisoning with preparations containing hydrocyanic acid. But it is not a constant appearance; for it was not observed in the seven Parisian epileptics. Neither is it peculiar; for death from carbonic acid has the same effect. I have remarked it six hours after death in a woman who died of cholera; and it has been observed in cases of death during the epileptic paroxysm."

*Treatment.*—This is too often hopeless on account of the rapidity of action of large and dangerous doses. The recommendation of emetics and enemata in these cases by Orfila is unintelligible. The necessity of arousing the depressed powers of the system suggested the employment of *ammonia;* but the experiments of Orfila tend to show this is only useful when instantly inhaled (1 part ammonia 12 water) and not swallowed. The remedy originated however with Mr. Murray in 1822. In the same year, too, Rianz proposed the inhalation of *Chlorine*, and Orfila has saved dogs when dying, by causing them to inspire water impregnated with a fourth of its volume of chlorine. *Cold Affusion* is another important means of arousing the failing powers. The coldest water should be procured, and poured upon the head and spinal column; keeping a bladder filled with ice also to the head, Mr. Banks has published a case (Ed. Journ. vol. 48), in which recovery followed the use of this means. Orfila seems to regard *venesection* only as proper during the ulterior treatment of the case; but others, in experiments upon animals, have found the opening the jugular vein a powerful means of relieving the engorged condition of the right side of the heart, and thus restoring its action.

"Few observations have hitherto been made on the *chemical antidotes* for hydrocyanic acid, or those substances which render it innoxious by converting it into an insoluble compound. It is plain that several probable antidotes of this kind exist. But toxicologists have been apparently deterred from trying them by the fearful rapidity with which the poison acts, and the consequent improbability that in practice such an antidote can be administered in time. It has lately been shown, however, by Messrs. Smith of this city, that the effects of a fatal dose may be warded off by the timely administration of the re-agents necessary for converting the acid into Prussian Blue. They found that if a solution of carbonate of potash, followed by a solution of the mixed sulphates of iron, be given to animals very soon after the administration of 30 drops of the Edinburgh medicinal acid, containing 3 per cent. real acid, recovery in general takes place, and sometimes little inconvenience seems to be sustained. The solutions they used were one of 144 gr. carb. pot. in 2 ℥ water, and another composed of ʒj½. sulph. protox. iron, together with ʒij. of the same salt converted into sulph. of sesquioxide by means of sulphuric and nitric acids in the usual way. About 52 minims of each solution will remove the whole acid contained in 100 gr. of Ed. medicinal acid; but for certainty, three or four times as much should be used—which may be done with perfect safety."

We need not say more in commendation of the two standard works whence we have borrowed so largely, save to repeat what a more particular examination impresses, that however necessary Professor Orfila's work is for reference, Dr. Christison's is a very superior guide for the general practitioner and student. We have not quoted from Dr. Guy's "Principles" as it is but a condensed (too concentrated for our taste) compilation, useful chiefly to those who are attending his class, but devoid of original matter.

The tenth edition of Mr. Stowe's well known "Toxicological Chart" has just been published, with the various additions which the progress of the science has enabled its author to make. He has not, however, inserted the new antidote for prussic acid which we have transcribed above, and we are not quite sure the *practical* utility of the Chart will be diminished by its omission. To young beginners a synopsis of this kind is indispensable.

In concluding this article, we may be allowed to give expression to a hope, that the time is not far distant, when some attempt will be made to place some restriction upon the *right of selling drugs*, and especially those of a *poisonous nature*. We are quite aware that murder and suicide by poisoning will not thus be wholly prevented, but we feel certain that much may be done for their diminution. Who can doubt that the present disgraceful facility in obtaining these dangerous substances has wonderfully aided the murderer in his diabolical projects? And who does not know that long premeditated suicide is the exceptive case, and that the deed would be often prevented, were the seeker after poison submitted to searching investigations, or compelled to reflect by reason of the delay which the difficulty of obtaining the instrument of destruction entailed upon him? The censure of the Coroner's Jury, unattended by any penal consequences, is of no use whatever; for it is quite certain that men, who for a few wretched pence, place these dangerous weapons in the hands of malice or ignorance, are far removed from any wholesome influence conveyed by a reprimand. Among other precautions, the imitation of the practice reported by Sir George Lefevre, as followed in Russia, of dispensing all dangerous compounds in peculiarly colored bottles or papers, would at least prevent accidents.

